# Supplementary material for: Impact of household size and co-resident multimorbidity on unplanned hospitalisation and transition to care home
Source: Nat Commun. 2025 Feb 17;16:1718. doi: 10.1038/s41467-025-56990-9 (PMC11836437; doi:10.1038/s41467-025-56990-9)
Supplement: Supplementary file 1 — Supplementary Information [file 41467_2025_56990_MOESM1_ESM.pdf]

Supplementary File. Impact of household size and co-resident multimorbidity on unplanned hospitalisation and transition to living in a care home: multilevel analysis of population data in Wales.

Table of Contents

Supplementary Table S1. Overview of included datasets, variables, and methods of linkage. 2

Supplementary Table S2. Baseline characteristics of child co-residents (not assessed for

Supplementary Information Panel S1. Methodology for ascertainment of multimorbidity in individuals and household coresidents. 6

Supplementary Table S3. Choice of long-term conditions in relation to those included by "Measuring multimorbidity in research: a Delphi consensus study" (Ho et al, 2022).[6] 7

Supplementary Table S4. List of included long-term conditions including explanation of rules and code-lists used to define these conditions. 9

Supplementary Table S5. Prevalence of conditions in the study cohort. 14

Supplementary Information Panel S2. Ascertainment of ethnicity. 15

Supplementary Table S6. Ascertainment of smoking status. 16

Supplementary Table S7. Ascertainment of alcohol consumption. 18

Supplementary Table S8. Ascertainment of body mass index (BMI). 19

Supplementary Table S9. Associations between all covariates with first unplanned hospitalisation for all variables. 20

Supplementary Table S10. Associations between all covariates with a transition to living in a care home for all variables. 21

Supplementary Table S11. Subgroup analyses 1: age stratification of unplanned hospitalisation

Supplementary Table S12. Subgroup analyses 2: age stratification of care home models. 23

Supplementary Table S13. Subgroup analyses 3: sex stratification of unplanned hospitalisation

Supplementary Table S14. Subgroup analyses 4: sex stratification of care home models. 25

Supplementary Table S15. Sensitivity analyses 5: household size and household co-resident mental-physical multimorbidity. 26

Supplementary Table S16. Sensitivity analyses 6: household size and household co-resident number of long-term conditions. 27

References 28

Supplementary Table S1. Overview of included datasets, variables, and methods of linkage.

All data were held in the SAIL Databank, which contains extensive anonymised health and administrative data about the population of Wales, accessible in anonymised form via a secure data sharing platform, all underpinned by the Information Governance model. All data within the SAIL Databank are treated in accordance with the Data Protection Act 2018 and are compliant with the General Data Protection Regulation. Researchers do not have access to personal identifiable data.

During the anonymisation process of data sources within the SAIL Databank, individuals are assigned an anonymised linking field (ALF) based on their National Health Service number, name, sex, date of birth, and residential address postcode.[2] Household units were identified by using the dwelling identification number (DWELLING_ID_PE). This is the household identifier derived from 2011 Census Wales data and not through probabilistic matching.

We restricted the sample to individuals registered with SAIL contributing General Practices (GP) (80% of GP practices and 83% of Welsh residents).[4] This was to ensure that primary and hospital inpatient health data were available, a method that we previously used to ascertain conditions using routinely collected data in the same dataset.[5]

Ensuring that participants were community-dwelling was done by excluding people living at an address registered as a care home and those living in households with seven or more inhabitants.

Dataset	Description	Variables within data source	Linkage
**Welsh Demographic Services Dataset (WDSD)**	Demographic dataset including the population register for SAIL Databank, used to create population subgroups for required dates and durations.	Age<br>Sex<br>Date of residence: start and end date used to identify individuals living within Wales at the study cross-section date (27 March 2011)<br>RALF for linkage with the CARE dataset (see below)	Anonymised linkage field (ALF_PE)
**Welsh Longitudinal General Practice Dataset (WLGP)**	Primary care dataset for GP practices contributing data to SAIL Databank in Wales. Data extracted form electronic clinical information systems including data regarding patient demographic and lifestyle characteristics, coding for conditions, clinical signs, symptoms, laboratory test results, and prescribed treatment.[4]	Registration with SAIL contributing GP practice<br>Quality metric for individual identification number<br>Ascertainment of ethnicity coding (see Supplementary Information Panel 1 for further information)<br>Ascertainment of conditions via diagnosis codes, laboratory biochemistry results, and prescribing (read version 2 – see Supplementary Table S3 for further information)<br>Ascertainment of smoking, alcohol, and body mass index (see Supplementary Tables S8-10 for a complete list of codes and methods for characterising these variables)	Anonymised linkage field (ALF_PE)
**Patient Episode Database for Wales (PEDW)**	NHS Wales hospital admission dataset comprising attendance and clinical information for all hospital admissions: including diagnoses and operations performed. Data are collected and coded at each hospital. After hospital discharge, the handwritten patient notes are transcribed by a clinical coder into medical terminology (ICD10 and codes).[8]	Ascertainment of conditions<br>Ascertainment of unplanned hospitalisation	Anonymised linkage field (ALF_PE)
**Office for National Statistics (ONS) 2011 Census Wales (CENW) Dataset**	Variables made available by the ONS regarding data collected from the person and dwelling datasets derived from the 2011 Census Wales.	Household identifier (the most reliable method of ascertaining residents living within the same residential address)	Person identification number (PERS_ID) and dwelling identification number. Linked to ALF via an intermediate linkage table
**Care homes dataset (CARE)**	List of residential anonymized linking fields for care homes	Used to ascertain individuals living in care homes.<br>Used to define:<br>1. Study cohort by excluding individuals living in a care home at the study cross-section date both at the study cross-section date, and	Linkage between residential anonymized linking fields RALF) and dwelling identification

		2. Care home admission outcome where an individual moved to a care home during the study follow-up.	number from CENW
**Annual District Deaths Extract (ADDE)**	Annual District Deaths Extract	Ascertainment of date of death.	Anonymised linkage field (ALF_PE)

Supplementary Table S2. Baseline characteristics of child co-residents (not assessed for multimorbidity).

Characteristics have been stratified according to household size. A total of 340,986 children were co-residents with study participants. Most children lived in three-or-more-person households. A smaller number of children lived with only one adult in two-person households. However, the proportion of children living with a sole adult coresident was substantially higher in areas of the lowest socioeconomic position (SEP): 30.7% in the lowest compared to 13.4% in the quintile of the highest SEP.

**Characteristics**	**Household size Number (%)**		**Total number of child co-residents Number (%)**
	**Two-person**	**Three-or-more person**	
	29,960 (8.8)	311,026 (91.2)	340,986
**Age in years (mean)**	8.7 (5.3)	9.1 (5.0)	9.1 (5.0)
**Sex**			
**Male**	15,304 (51.1)	159,800 (51.4)	175,104 (51.3)
**Female**	14,656 (48.9)	151,226 (48.6)	165,882 (48.6)
**Ethnicity**			
**White**	27,813 (92.8)	290,771 (93.5)	318,584 (93.4)
**Mixed**	1,005 (3.4)	6,301 (2.0)	7,306 (2.1)
**Asian**	357 (1.2)	8,523 (2.7)	8,880 (2.6)
**Black**	173 (0.6)	1,653 (0.5)	1,826 (0.5)
**Other**	95 (0.3)	1,556 (0.5)	1,651 (0.5)
**Missing**	517 (1.7)	2,222 (0.7)	2,739 (0.8)
**WIMD**			
**1 (lowest SEP)**	9,185 (30.7)	66,558 (21.4)	75,743 (22.2)
**2**	7,198 (24)	63,190 (20.3)	70,388 (20.6)
**3**	5,567 (18.6)	60,608 (19.5)	66,175 (19.4)
**4**	4,004 (13.4)	53,898 (17.3)	57,902 (17)
**5 (highest SEP)**	4,006 (13.4)	66,772 (21.5)	70,778 (20.8)

Supplementary Information Panel S1. Methodology for ascertainment of multimorbidity in individuals and household coresidents.

Main analyses examined associations when describing multimorbidity as two or more conditions (multimorbidity) and at least one mental and one physical health condition (mental-physical multimorbidity). These definitions were chosen because the majority of multimorbidity research literature adopts the multimorbidity definition,[1] and results from our previous study using data from a similar population cohort that finds that individuals living in the most deprived areas had mental-physical multimorbidity 40-45 years younger than those living in the least deprived areas.[3] Conditions considered in counts of multimorbidity were derived from a recent Delphi study including professionals interested in multimorbidity and people living with multimorbidity, resulting in a list of 47 long-term conditions recommended for use in multimorbidity research[6] (Supplementary Table S3). We have previously tested this list of conditions within a similar population (the English National Health Service) and found that it provides stable estimates of multimorbidity prevalence.[7]

Counts of conditions were ascertained on the study cross-section date and were defined from primary care data using Read version 2 diagnosis codes, prescribing, and laboratory data, and from hospital inpatient data using ICD-10 diagnosis codes. We used both primary care and hospital inpatient data to generate robust ascertainment of conditions as examined in our previous study using the same dataset.[5] Ascertainment of each condition included rules to ensure only active conditions were counted where a separate set of criteria were determined for each condition (Supplementary Table S4). For example, we ensured that commonly remitting conditions such as asthma, depression, and anxiety were active at the time under evaluation in the study by using methods such as time-limited look-back for diagnosis codes and prescribing data.

Supplementary Table S3. Choice of long-term conditions in relation to those included by "Measuring multimorbidity in research: a Delphi consensus study" (Ho et al, 2022).[6]

Description of the rationale for inclusion or exclusion for each condition, and aggregation and allocation to body system.

Body system as defined by Delphi study	Delphi "always include" conditions	Delphi "usually include" conditions	Inclusion (including aggregation into higher-level condition) or exclusion (and rationale)
Cardiovascular system	Stroke	-	Included (as stroke AND transient ischaemic attack combined)
	Coronary artery disease	-	Included
	Heart failure	-	Included
	Peripheral arterial disease	-	Included
	-	Heart valve disorders	Included
	-	Arrythmia	Included
	-	Venous thromboembolic disease	Included
	-	Aneurysm	Included
		Hypertension	Included
Metabolic and endocrine disease	Diabetes	-	Included
	Addison's disease	-	Included
	Cystic fibrosis	-	Included
	-	Thyroid disorders	Included
Respiratory disease	Chronic obstructive pulmonary disease	-	Included
	Asthma	-	Included
	-	Bronchiectasis	
Neurological disease	Parkinson's disease	-	Included
	Epilepsy	-	Included
	Multiple sclerosis	-	Included
	Paralysis	-	Included
		Transient ischaemic attack	Included (as stroke AND transient ischaemic attack combined)
	-	Peripheral neuropathy	Included
	-	Chronic primary pain	Excluded: it was decided to exclude based on CPRD @ Cambridge prescribing code list (https://www.phpc.cam.ac.uk/pcu/research/research-groups/crmh/cprd_cam/codelists/v11/) where listed analgesics could result in double counting of conditions. It was deemed difficult to avoid with use of a broad range of analgesic medications used for mild to moderate pain, for example osteo-arthritis for paracetamol, codeine-containing compounds, or non-steroidal anti-inflammatory drugs, and cancer for strong analgesics such as morphine
Cancer	Solid organ cancers	-	Included (as cancer)
	Haematological cancers (included as cancer)	-	Included (as cancer)
	Metastatic cancers (included as cancer)	-	Included (as cancer)
	-	Melanoma (included as cancer)	Included (as cancer)
	-	Cerebral tumours that can cause disability	Excluded: it was decided to exclude based on difficulty in defining this population using code lists applied to routinely collected data

Mental and behavioural disorder	Dementia	-	Included
	Schizophrenia	-	Included
		Depression	Included
		Bipolar disorder	Included
		Drug or alcohol misuse	Included
		Eating disorder	Included
		Autism	Included
		Post-traumatic stress disorder	Included
Musculoskeletal disease	Connective tissue disease		Included
		Osteoarthritis	Included
	-	Long term musculoskeletal problems due to injury	Excluded: our group decided to exclude due to difficulty in accurately coding and identifying which conditions cause long-term impairment
	-	Osteoporosis	Included
		Gout	Included
Urogential disorder	Chronic kidney disease		Included
	End stage kidney disease (included as chronic kidney disease)	-	Included
	-	Endometriosis	Included
		Chronic urinary tract infection	Excluded: it was decided to exclude due to difficulty in accurately coding and identifying which conditions cause long-term impairment. Using antimicrobial prescribing other than trimethoprim and nitrofurantoin also presents difficulties because these agents are not specific to infections of the urinary tract and broader spectrum antimicrobial agents could be used to treat acute soft tissue and respiratory infections
Haematological disorder	-	Anaemia	Included
Eye disease	-	Vision impairment that cannot be corrected	Included
Ear disease	-	Hearing impairment that cannot be corrected	Included
	-	Meniere's disease	Included
Infectious disease	HIV	-	Excluded: not accessible in SAIL Databank
		Chronic Lyme disease	Excluded: it was decided to exclude due to difficulty in accurately coding and identifying which conditions cause long-term impairment
	-	Tuberculosis	Included
		Post-acute covid-19 (study pre-2020)	Study period pre-2020
Congenital disease	-	Congenital disease and chromosomal abnormalities	Included
Digestive disease	Chronic liver disease	-	Included
	Inflammatory bowel disease	-	Included
	-	Chronic pancreatitis	Included
	-	Peptic ulcer	Included

Supplementary Table S4. List of included long-term conditions including explanation of rules and code-lists used to define these conditions.

Choice of the 47 conditions was based on results of a recent Delphi consensus study recommending those to include in measurement of multimorbidity,[6] and tested for stability in prevalence estimates.[9] The primary care dataset was the Welsh Longitudinal General Practice Dataset (WLGP), and the hospital inpatient dataset was Patient Episode Database for Wales (PEDW). Phenotype definition and look-back duration for the codes defining each of the conditions followed rules defined by Barnett et al[10] where possible and applied to examine condition prevalence ascertaining codes from primary care, hospital inpatient, and linked primary care to hospital inpatient records.[5] For the remaining conditions, inclusion criteria were agreed through discussion between authors CM, SWM, and BG. In certain cases, look-back durations varied within conditions to reflect the impact living with the condition was likely to have on an individual. For example, anaemia was defined as a relevant code ever recorded for aplastic anaemia, sickle cell anaemia, thalassaemia (conditions that are either life-long or life-threatening), but as a relevant code dated in the 12-months prior to the study cross-section date for iron-, B12- or folate-deficient anaemias (conditions that are more likely to be transient), with the results of both combined into a single variable defining the presence of ‘anaemia’ on the 27 March 2011. Unless the look-back duration was specifically stipulated, for example 1-year for asthma clinical codes, codes present between 1st January 2000 and the study cross-section date of 27 March 2011 were used for both primary care and hospital inpatient data. This approach was taken to avoid relative over ascertainment of primary care codes because historic codes are present for lifetime records that have been transcribed into the electronic record in the primary care data source, but the first electronic records in the hospital inpatient dataset held within PEDW began on 1st April 1995. Code lists used to define conditions were those created by Kuan et al[11] available on the HDR UK Phenotype Library,[12] and de novo code lists created specifically by the authors of this study where required (detailed in Additional File 2). We adapted prescribing code lists from the Cambridge Multimorbidity Score by Payne et al to qualify conditions that resolve as ‘active’ on 27 March 2011 (e.g., asthma and epilepsy).[13]

Condition name	Implementation rules	Code list Read v2*,**	Code list ICD-10*,**
Addison's disease	Read v2 or ICD-10 code ever recorded	A176., C154., C1540, C1541, C1540, C154z	E271, E272, E271, E274
Alcohol and substance misuse	Read v2 or ICD-10 code ever recorded	c945 - alcohol misuse,<br>c1594 - substance misuse	c947 - alcohol misuse,<br>1596 - substance misuse
Anaemia	Read v2 or ICD10 code 12-month look back (iron deficiency, B12 deficiency, folate deficiency), Read v 2 or ICD10 code ever recorded (aplastic anaemia, sickle cell anaemia, thalassaemia)	c831 – iron deficiency anaemia, c979 - aplastic anaemia, c1013 - B12 deficiency anaemia, c1174 - folate deficiency anaemia, c1556 - sickle cell anaemia, c1603 - thalassaemia	c833 - iron deficiency anaemia, c981 - aplastic anaemia, c1015 - B12 deficiency anaemia, c1176 - folate deficiency anaemia, c1558 - sickle cell anaemia, c1602 - thalassaemia
Aneurysm	Read v2 or ICD-10 code ever recorded	c783 - abdominal aortic aneurysm	c785 - abdominal aortic aneurysm
Anxiety	Read v2 or ICD-10 code in the previous 12-months OR 4 or more anxiolytic/hypnotic prescriptions in the previous 12-months	c976 - anxiety	c978 - anxiety
Arrythmia	Read v2 or ICD-10 code ever recorded	c789 - atrial fibrillation, c915 – supraventricular tachycardia, c1010 – atrioventricular block complete<br>c1553 - sick sinus syndrome, c1615 - trifasicular block	c791 - atrial fibrillation, c917 – supraventricular tachycardia, c1012 – atrioventricular block complete, c1555 - sick sinus syndrome, c1617 - trifasicular block
Asthma	Read v2 or ICD-10 code ever recorded AND any prescription in the last 12 months AND no Read v2 or ICD-10 Chronic Obstructive Pulmonary Disease code ever recorded	c2418 - asthma	c994 - asthma
Autism	Read v2 or ICD-10 code ever recorded	c995 - autism	c997 - autism
Bipolar affective disorder	Read v 2 or ICD-10 code ever coded OR lithium ever prescribed	c793 - bipolar	c795 - bipolar
Bronchiectasis	Read v2 or ICD-10 code ever recorded	c1045 - bronchiectasis	c1047 - bronchiectasis
Coronary artery disease	Any Read v2 or ICD-10 code ever recorded	c802 – coronary heart disease, c1296 – myocardial infarction, c1588 - stable angina, c1631 - unstable angina	c804 – coronary heart disease, c1298 – myocardial infarction, c1590 - stable angina, c1633 – unstable angina, c1590 - stable angina,<br>c1633 - unstable angina
Cancer (Delphi: Solid organ cancers, haematological cancer, metastatic cancer, melanoma)	First Read v2 or ICD-10 code in the previous 12-months	c846 – myelodysplastic, c858 - non-Hodgkin's lymphoma, c1218 - Hodgkin's lymphoma, c1258 – leukaemia, c1361 - primary myeloma, c1415 - primary melanoma, c1385 - primary biliary, c1388 - primary bladder, c1391 - primary bone, c1397 - primary brain, c1400 - primary breast, c1403 - primary cervical, c1406 - primary kidney, c1409 - primary liver, c1412 - primary lung, c1418 - primary mesothelioma, c1421 -	c848 – myelodysplastic, c860 - non-Hodgkin's lymphoma, c1220 - Hodgkin's lymphoma, c1260 – leukaemia, c1363 - primary myeloma, c1417 - primary melanoma, c1387 - primary biliary, c1390 - primary bladder, c1393 - primary bone, c1399 - primary brain, c1402 - primary breast, c1405 - primary cervical, c1408 - primary kidney, c1411 - primary liver, c1414 - primary lung, c1420 - primary mesothelioma, c1423 -

		primary multiple, c1424 - primary oesophageal, c1430 - primary other, c1433 - primary ovarian, c1436 - primary pancreatic, c1439 - primary prostate, c1445 - primary stomach, c1448 - primary testicular, c1454 - primary thyroid, c1457 - primary uterine, c1513 - secondary lymph nodes, c1516 - secondary adrenal, c1519 - secondary bone, c1522 - secondary bowel, c2065 - bowel cancer primary or secondary, c1525 - secondary brain, c1528 - secondary liver, c1531 - secondary lung, c1540 - secondary peritoneum, c1543 - secondary pleura, c1537 - secondary other	primary multiple, c1426 - primary oesophageal, c1432 - primary other, c1435 - primary ovarian, c1438 - primary pancreatic, c1441 - primary prostate, c1447 - primary stomach, c1450 - primary testicular, c1450 - primary thyroid, c1459 - primary uterine, c1515 - secondary lymph nodes, c1518 - secondary adrenal, c1521 - secondary bone, c1524 - secondary bowel, c1527 - secondary brain, c1530 - secondary liver, c1533 - secondary lung, c1542 - secondary peritoneum, c1545 - secondary pleura, c1539 – secondary other
Cystic fibrosis	Any Read v2 or ICD-10 or Read v2 code ever recorded<br>AND NO bronchiectasis Read v2 or ICD-10 code ever recorded	C799 – cystic fibrosis	c801 - cystic fibrosis
Chromosomal abnormalities†	Any Read v2 or ICD-10 code ever recorded	PJ00., PJ01., PJ01., PJ0.., PJ0.., PJ0z., PJ0z., PJ0.., PJ02., PJ02., PJ20., PJ21., PJ21., PJ2.., PJ2z., PJ2z., PJ22., PJ10., PJ11., PJ11., PJ1.., PKyz0, PJ1z., PJ1z., PJ12., PJ50w, PJ50x, PJ50x, PJ510, PJ511, PJ520, PJ521, PJ523, PJ524, PJ513, PJ513, PJ515, PJ515, PJ512, PJ503, PJ514, PJ514, PJ500, PJ501, PJ502, PJ503, PJ504, PJ505, PJ506, PJ0.., PJ508, PJ50., PJ51., PJ51z, PJz3., PJ50z, PyuA0, PJ507, PJ50y, PJ52., PJ52z, PJ36., PJ370, PJ37., PJ37z, PJ37., PJ38., PJ32., PJ31., PJ338, PJ339, PKyz7, PKyz5, PKyz7, PKyz5, PJ330, PJ30., PJz2., PJ3z., PJ30.11, PyuA1, PJ33., PJ33z, PJ3y., PJ331, PJ332, PJ331, PJ332, PJ332, PJ331, PJ333, PJ336, PJ337, PJ334, PJ34., PJ35., PJ3.., PyuA2, PJ37., PJ531, PJ632, PJ633. PJ60., PJy10, PJ635, PJ630, PJ636, PJ634, PJ63., PJ63z, PJ63z, PyuA5, PJ636, PJ63z, PJ6.., PJ6z., PJ64., PJ64z, PJ62., PJ631, PJy2., PJy2., PJy4., PJy5..PJ640, PyuA6, PJy12, PJyy0, PJyy1, PJyy4, PJyy2, F1y0., PyuAB, PJy1z, PJy1., PJy13, PJyy., PyuAD	Q968, Q969, Q97, Q970, Q971, Q972, Q973, Q978, Q979, Q980, Q99, Q990, Q991, Q992, Q998, Q999, Q87, Q870, Q871, Q872, Q873, Q874, Q875, Q878, Q878

Chronic kidney disease	Any coding as per PC implementation rules OR ICD-10 code ever recorded	c811 - end stage renal disease c2035 - chronic kidney disease	c813 - end stage renal disease c2846 - chronic kidney disease
Chronic liver disease	Read v2 or ICD-10 code ever recorded	c998 - autoimmune liver disease, c1373 - portal HTN, c1082 - liver fibrosis, sclerosis, and cirrhosis, c1265 - alcoholic liver disease, c1645 - oesophageal varices, c1073 – chronic viral hepatitis	c1000 - autoimmune liver disease, c1375 - portal HTN, c1084 - liver fibrosis, sclerosis, and cirrhosis, c1267 - alcoholic liver disease, c1647 - oesophageal varices, c1075 chronic viral hepatitis
Chronic obstructive pulmonary disease	Read v2 or ICD-10 code ever recorded	c2748 – chronic obstructive pulmonary disease	c2746 - chronic obstructive pulmonary disease
Connective tissue disorders	Read v2 or ICD-10 code ever recorded	c815 – giant cell arteritis, c887 – polymyalgia rheumatica, c905 – rheumatoid arthritis, c912 – systemic lupus erythematosus, c961 - ankylosing spondylosis, c1249 - juvenile arthritis, c1565 – Sjogren syndrome, c890 - psoriatic arthritis	c817 – giant cell arteritis, c889 – polymyalgia rheumatica, c907 – rheumatoid arthritis, c914 – systemic lupus erythematosus, c963 - ankylosing spondylosis, c1251 juvenile arthritis, c1567 Sjogren syndrome, c892 psoriatic arthritis
Dementia	Read v2 or ICD-10 code ever recorded	c2777 - dementia	c2773 - dementia
Depression	Read v2 or ICD-10 code in the previous 12-months OR 4 or more antidepressant prescriptions in the previous 12-months	c1111 - depression	c1113 - depression
Diabetes	Read v2 or ICD-10 code ever recorded	c1120 – diabetes, c1128 - diabetes neurological complications, c1117 - diabetes eye complications	c1122 – diabetes, c1130 - diabetes neurological complications, c1119 - diabetes eye complications
Eating disorder	Read v2 or ICD-10 code ever recorded	C3252 - eating disorder	c2935 - eating disorder
Endometriosis	Any Read v2 or ICD-10 code ever recorded AND age < 55 years	c1145 - endometriosis	c1147 - endometriosis
Epilepsy	Read v2 or ICD-10 code ever coded AND any antiepileptic (excluding gabapentinoids) prescription in the previous12 months	c1154 - epilepsy	c1156 - epilepsy
Gout	Read v2 or ICD-10 code ever recorded	c1191 - gout	c1193 - gout
Hearing impairment	Read v2 or ICD-10 code ever recorded	c1102 - hearing loss	c1104 - hearing loss
Heart failure	Read v2 or ICD-10 code ever recorded	c1206 - heart failure	c1208 - heart failure
Heart valve disorders	Read v2 or ICD-10 code ever recorded	c908 - rheumatic heart valve, c1289 - multiple heart valve disorder, c1308 - nonrheumatic aortic valve disorder, c1311 - nonrheumatic mitral valve disorder	c910 - rheumatic heart valve disorder, c1291 - multiple heart valve disorder, c1310 - nonrheumatic aortic valve disorder, c1313 - nonrheumatic mitral valve disorder
Hypertension	Read v2 or ICD-10 code ever recorded	c1227 - hypertension	c1229 - hypertension
Inflammatory bowel disease	Read v2 or ICD-10 code ever recorded	c1096 - Crohn's disease, c1621 - ulcerative colitis	c1098 - Crohn's disease, c1623 - ulcerative colitis

Meniere’s disease	Read v2 or ICD-10 code ever recorded	c1279 - Meniere's disease	c1281 - Meniere's disease
Multiple sclerosis	Read v2 or ICD-10 code ever recorded	c855 – Multiple Sclerosis	C857 – Multiple sclerosis
Osteoarthritis	Read v2 or ICD-10 code ever recorded	c861 - osteoarthritis	c863 - osteoarthritis
Osteoporosis	Read v2 or ICD-10 code ever recorded	c1326 - osteoporosis	c1328 - osteoporosis
Peripheral arterial disease	Read v2 or ICD-10 code ever recorded	c1349 – peripheral arterial disease	c1351 - peripheral arterial disease
Pancreatitis (chronic)	Read v2 or ICD-10 code ever recorded	14CG., J671.	K861, K860
Paralysis	Read v2 or ICD-10 code ever recorded	F2411, F141., F241., F2410, F2300, F240.. F2401, F2400, F232., F232.. F240., F242.. F230., F230z, F2301	G82, G830, G808, G801, G803
Parkinson’s disease	Read v2 or ICD-10 code ever recorded	c896 – Parkinson’s disease	c898 - Parkinson’s disease
Peptic ulcer	Read v2 or ICD-10 code ever recorded	c1624 - peptic ulcer	c1626 - peptic ulcer
Peripheral neuropathy	Read v2 or ICD-10 code ever recorded	c1346 - peripheral neuropathy	c1348 - peripheral neuropathy
Post-traumatic stress disorder	Read v2 or ICD-10 code ever recorded	E2831, E29y1, Eu431, Eu433, Eu434, ZS7C7	F431
Schizophrenia	Read v2 or ICD-10 code ever recorded	c1503 - schizophrenia	c1503 - schizophrenia
Stroke and transient ischaemic attack	Read v2 or ICD-10 code ever recorded	c834 - intracerebral haemorrhage, c837 - ischaemic stroke, c918 - NOS stroke, c921 - subarachnoid haemorrhage, c921, c927 – transient ischaemic attack	c836 - intracerebral haemorrhage, c839 - ischaemic stroke, c920 - NOS stroke, c923 - subarachnoid haemorrhage, c929 transient ischaemic attack
Tuberculosis	Read v2 of ICD-10 code in the previous 5 years	c924 - tuberculosis	c926 - tuberculosis
Thyroid disorders	Read v2 or ICD-10 code ever recorded	C1609 - thyroid	C1611 - thyroid
Visual impairment	Read v2 or ICD-10 code ever recorded	c1041 - visual impairment and blindness	c1043 - visual impairment and blindness
Venous thromboembolic disease	Read v2 or ICD-10 code in previous 12-months or >1 code ever	c880 – pulmonary embolism, c1657 - deep vein thrombosis	c882 – pulmonary embolism, c1659 – deep vein thrombosis

NA = not applicable, condition not included in Barnett et al[10] analysis
*Read v2 codes truncated to 5-digits for compatibility with SAIL Databank.
**Code lists from the HDR UK Phenotype Library are formatted as cXXX (e.g., c882 is pulmonary embolism) where they are called ‘concepts’ and can be downloaded by searching for the code list/concept number at https://phenotypes.healthdatagateway.org/concepts/?
†Available at OpenSAFELY https://github.com/opensafely/hdruk-os-covid-paeds/commit/6295c353125577798fafe9afa25d882a1b911200

Supplementary Table S5. Prevalence of conditions in the study cohort.

**Condition**	**Number (%)**<br>N = 1,472,185
Addison’s disease	832 (0.1)
Alcohol and substance misuse	50,240 (3.4)
Anaemia	19,262 (1.3)
Aneurysm	4,084 (0.3)
Anxiety	68,500 (4.7)
Arrythmia	58,582 (4.0)
Asthma	134,052 (9.1)
Autism	1,662 (0.1)
Bipolar affective disorder	9,963 (0.7)
Bronchiectasis	6,416 (0.4)
Coronary artery disease	109,264 (7.4)
Cancer	37,136 (2.5)
Cystic fibrosis	595 (0.0)
Chromosome	3,908 (0.3)
Chronic kidney disease	121,430 (8.2)
Chronic liver disease	9,748 (0.7)
Chronic obstructive pulmonary disease	56,789 (3.9)
Connective tissue disorder	44,483 (3.0)
Dementia	7,744 (0.5)
Depression	171,960 (11.7)
Diabetes	106,836 (7.3)
Eating disorder	6,832 (0.5)
Endometriosis	15,186 (1.0)
Epilepsy	15,245 (1.0)
Gout	52,776 (3.6)
Heart failure	31,678 (2.2)
Heart valve	26,665 (1.8)
Hypertension	362,078 (24.6)
Inflammatory bowel disease	16,535 (1.1)
Meniere’s disease	18,586 (1.3)
Multiple sclerosis	3,703 (0.3)
Osteoarthritis	208,904 (14.2)
Osteoporosis	64,486 (4.4)
Peripheral arterial disease	27,513 (1.9)
Pancreatitis	2,089 (0.1)
Paralysis	1,880 (0.1)
Parkinson’s disease	4,133 (0.3)
Peptic ulcer disease	39,364 (2.7)
Peripheral neuropathy	35,840 (2.4)
Post-traumatic stress disorder	5,475 (0.4)
Schizophrenia	10,025 (0.7)
Stroke and transient ischaemic attack	50,729 (3.4)
Thyroid disorders	92,112 (6.3)
Tuberculosis	267 (0.0)
Vision loss	14,745 (1.0)
Venous thromboembolism	59,354 (4.0)

Supplementary Information Panel S2. Ascertainment of ethnicity.

Ethnicity was primarily identified using Wales Census 2011 – Welsh Records (CENW) using the categories made available by National Statistics Wales.[14] Within this dataset, ethnicity is coded as Asian or Asian British, Black, African, Caribbean or Black British, Mixed or multiple ethnic groups, White, Other ethnic group. Where not present within Wales Census data, ethnicity codes present in routinely collected data available in Welsh Longitudinal General Practice Dataset (WLGP) and Patient Episode Dataset for Wales (PEDW) were used.[15] It is acknowledged that ethnic categories used in official statistics can be arbitrary having been selected primarily for pragmatic reasons[16], however building understanding of risk factors for adverse outcomes for those based on ethnicity is important given ethnic inequalities in clinical outcomes.[17]

Supplementary Table S6. Ascertainment of smoking status.

Smoking status was ascertained through ascertainment of Read v2 and EMIS codes in the primary care dataset, the Welsh Longitudinal General Practice Dataset (WLGP). First, ascertainment of smoking quantity codes indicating that an individual smokes were recorded within the 5 years prior to study cross-section date: “Ex-smoker”, “Light smoker”, “Moderate smoker”, “Heavy smoker”. Where one of these codes was not present, a search for codes recording “Never smoked” with any time lookback was performed and where present the person was coded as such.

Phase One. Ascertainment of smoking quantity codes within the 5-years prior to study cross-section date.

**Code**	**Code description**	**Smoking status**
Y6628	Ex-smoker	Ex-smoker
Y6627	Ex pipe smoker	Ex-smoker
XE0op	Ex-cigarette smoker amount unknown	Ex-smoker
XE0on	Ex-very heavy cigarette smoker (40+/day)	Ex-smoker
XE0om	Ex-heavy cigarette smoker (20-39/day)	Ex-smoker
XE0ol	Ex-moderate cigarette smoker (10-19/day)	Ex-smoker
XE0ok	Ex-light cigarette smoker (1-9/day)	Ex-smoker
XE0oj	Ex-trivial cigarette smoker (<1/day)	Ex-smoker
XaQ8V	Ex roll-up cigarette smoker	Ex-smoker
Xa1bv	Ex-cigarette smoker	Ex-smoker
Ub1na	Ex-smoker	Ex-smoker
EGTON327	Past smoker	Ex-smoker
EGTON327	Past smoker	Ex-smoker
EGTON322	Ex-Cigarette Smoker	Ex-smoker
EGTON322	Ex-Cigarette Smoker	Ex-smoker
EGTON1028	Ex-smoker NOS	Ex-smoker
EGTON1028	Ex-smoker NOS	Ex-smoker
EGTON1027	Ex- Rolled Tobacco Smoker	Ex-smoker
EGTON1027	Ex- Rolled Tobacco Smoker	Ex-smoker
137S.	Ex-smoker	Ex-smoker
137O.	Ex-cigar smoker	Ex-smoker
137l.	Ex roll-up cigarette smoker	Ex-smoker
137K0	Recently stopped smoking	Ex-smoker
137K.	Stopped smoking	Ex-smoker
137j.00	Ex-cigarette smoker	Ex-smoker
137F.	Ex-smoker - amount unknown	Ex-smoker
137B.	Ex-very heavy smoker (40+/day)	Ex-smoker
137A.	Ex-heavy smoker (20-39/day)	Ex-smoker
^ESCTEX801757	Ex-smoker for more than 1 year	Ex-smoker
^ESCTEX549603	Ex-cigarette smoker amount unknown	Ex-smoker
^ESCT1172048	Ex-trivial smoker (<1/day)	Ex-smoker
^ESCT1172045	Ex-light smoker (1-9/day)	Ex-smoker
^ESCT1172042	Ex-moderate smoker (10-19/day)	Ex-smoker
^ESCT1172039	Ex-heavy smoker (20-39/day)	Ex-smoker
^ESCT1172035	Ex-smoker amount unknown	Ex-smoker
^ESCT1165113	Ex-smoker for less than 1 year	Ex-smoker
1379	Ex-moderate smoker (10-19/day)	Ex-smoker
1378	Ex-light smoker (1-9/day)	Ex-smoker
1377	Ex-trivial smoker (<1/day)	Ex-smoker
Y7467	Light smoker - 1-9 cigs/day	Light smoker
XE0oi	Trivial cigarette smoker (less than one cigarette/day)	Light smoker
XagO3	Occasional tobacco smoker	Light smoker
Ub1tS	Light cigarette smoker	Light smoker
Ub1tR	Occasional cigarette smoker	Light smoker

EGTONGR11	Grade B light smoker (1-10/day)	Light smoker
EGTONGR11	Grade B light smoker (1-10/day)	Light smoker
1372-1	Occasional smoker	Light smoker
^ESCTTR500314	Trivial cigarette smoker	Light smoker
^ESCTOC549595	Occasional cigarette smoker (less than one cigarette/day)	Light smoker
^ESCTLI500315	Light cigarette smoker	Light smoker
^ESCT1190499	Occasional cigarette smoker	Light smoker
1373	Light smoker - 1-9 cigs/day	Light smoker
1372.11	Occasional smoker	Light smoker
Y7677	Moderate smoker - 10-19 cigs/d	Moderate smoker
Ub1tT	Moderate cigarette smoker	Moderate smoker
EGTONGR12	Grade C moderate smoker (11-20/day)	Moderate smoker
^ESCTMO500316	Moderate cigarette smoker	Moderate smoker
^ESCTMO341910	Moderate smoker (20 or less per day)	Moderate smoker
1374	Moderate smoker - 10-19 cigs/d	Moderate smoker
Y9843	Very heavy smoker - 40+cigs/d	Heavy smoker
Y7110	Heavy smoker - 20-39 cigs/day	Heavy smoker
Ub1tW	Chain smoker	Heavy smoker
Ub1tV	Very heavy cigarette smoker	Heavy smoker
Ub1tU	Heavy cigarette smoker	Heavy smoker
EGTONGR13	Grade D heavy smoker (>20 Day)	Heavy smoker
EGTONGR13	Grade D heavy smoker (>20 Day)	Heavy smoker
^ESCTVE500318	Very heavy cigarette smoker	Heavy smoker
^ESCTHE500317	Heavy cigarette smoker	Heavy smoker
^ESCTHE342222	Heavy smoker (over 20 per day)	Heavy smoker
1376	Very heavy smoker - 40+cigs/d	Heavy smoker
1375	Heavy smoker - 20-39 cigs/day	Heavy smoker

Phase Two. Ascertainment of never smoked codes ever recorded and applied to people without a smoking quantity code recorded in the 5-years prior to the study cross-section date.

**Code**	**Code description**	**Smoking status**
^ESCTNE796504	Never smoked any substance	Never smoked
^ESCTNE549592	Never smoked	Never smoked
1371	Never smoked tobacco	Never smoked
XE0oh	Never smoked tobacco	Never smoked

Supplementary Table S7. Ascertainment of alcohol consumption.

Alcohol consumption was ascertained through ascertainment of Read v2 and EMIS codes in the primary care register, the Welsh Longitudinal General Practice Dataset (WLGP). Alcohol consumption was categorised through ascertainment of Read v2 and EMIS codes in the WLGP database. Read v2 codes with associated values of alcohol consumption per day and per week were included. Categories of alcohol consumption were transformed from recorded consumption values into the following categories: 0 = ‘Zero’, 1-14 units = ‘Light (1-2 u/day)’, 15-42 = ‘Moderate (3-6u/day)’, 43-63 = ‘Heavy (7-9u/day)’, 64+ = ‘Very heavy (>9u/day)’. Where codes were available, the mist recent recorded value for each code was used.

**Code**	**Code description**	**Alcohol consumption**
1365	Heavy drinker - 7-9u/day	Heavy (7-9u/day)
136P.	Heavy drinker	Heavy (7-9u/day)
1363	Light drinker - 1-2u/day	Light (1-2u/day)
136J.	Social drinker	Light (1-2u/day)
136N.	Light drinker	Light (1-2u/day)
1364	Moderate drinker - 3-6u/day	Moderate (3-6u/day)
136O.	Moderate drinker	Moderate (3-6u/day)
EGTONGR8	Grade C 21-49 units/week	Moderate (3-6u/day)
1362	Drinks rarely	Trivial (<1u/day)
^ESCTVE453323	Very heavy drinker - greater than 9 units/day	Very heavy (>9u/day)
1366	Very heavy drinker - >9u/day	Very heavy (>9u/day)
136Q.	Very heavy drinker	Very heavy (>9u/day)
EGTONGR9	Grade D 50 or more units/week	Very heavy (>9u/day)
^ESCT1362881	Lifetime non-drinker of alcohol	Zero
^ESCTDO412035	Does not drink alcohol	Zero
^ESCTEX384349	Ex-drinker	Zero
^ESCTEX384349	Ex-drinker	Zero
^ESCTLI497791	Lifetime non-drinker	Zero
^ESCTNE412034	Never drinks	Zero
1361	Non-drinker alcohol	Zero
1367	Stopped drinking alcohol	Zero
136A.	Ex-trivial drinker (<1u/day)	Zero
136B.	Ex-light drinker - (1-2u/day)	Zero
136C.	Ex-moderate drinker - (3-6u/d)	Zero
136D.	Ex-heavy drinker - (7-9u/day)	Zero
136E.	Ex-very heavy drinker-(>9u/d)	Zero
136M.	Current nondrinker	Zero
^ESCTNO412037	Non - drinker alcohol	Zero
^ESCTAL845878	Daily alcohol intake	Use associated value
^EMISNQAU120	Daily alcohol intake	Use associated value
EMISNQAU130	Daily alcohol intake	Use associated value
136..	Weekly alcohol intake	Use associated value
136v.	Weekly alcohol intake	Use associated value
136Z.	Weekly alcohol intake	Use associated value
^ESCT1414751	Weekly alcohol intake	Use associated value
ESCTAL453314	Weekly alcohol intake	Use associated value
^ESCTAL498716	Weekly alcohol intake	Use associated value

Supplementary Table S8. Ascertainment of body mass index (BMI).

Body mass index (BMI) was calculated through ascertainment of Read v2 codes found in the WLGP dataset: directly from BMI values where coded using “22K..” or calculated from height “229..” and weight “22A..” where coded. Lookback was constrained to 5 years due to the potential variation of BMI across the life course. The most recent value of either BMI value or weight (and subsequent calculation of BMI) was used for each individual. Body mass index defined according to the National Institute for Clinical Excellence recommendations (https://cks.nice.org.uk/topics/obesity/#:~:text=Different%20weight%20classes%20are%20defined,%E2%80%9334.9%20kg%2Fm2.)

**Description**	**Read v2 code**
Body mass index	22K..
Height	229..
Weight	22A..

Supplementary Table S9. Associations between all covariates with first unplanned hospitalisation for all variables.

Event rate per 1000 person-years and hazard ratios (HRs) with 95% confidence intervals (95%CIs) for unadjusted, partially adjusted, and fully adjusted models. The partially adjusted model incorporates age group, sex, and socioeconomic position. The fully adjusted model incorporates age group, sex, socioeconomic position, number of long-term conditions, smoking, and alcohol consumption.

**Covariate**	**Event rate/1000 person years**	**Unadjusted HR (95% CI)**	**Partially adjusted HR (95% CI)** *	**Fully adjusted HR (95% CI)** **
Household size & co-resident multimorbidity				
Lives alone	91.96 (91.41-92.51)	Reference (1.00)	Reference (1.00)	Reference (1.00)
2-person household co-resident no multimorbidity	53.85 (53.45-54.24)	0.55 (0.54-0.55)	0.81 (0.80-0.82)	0.89 (0.89-0.90)
2-person household co-resident multimorbidity	85.46 (84.89-86.04)	0.88 (0.88-0.89)	0.90 (0.89-0.90)	0.91 (0.90-0.92)
≥3-person household co-resident no multimorbidity	39.88 (39.6-40.17)	0.37 (0.37-0.37)	0.76 (0.75-0.76)	0.87 (0.86-0.88)
≥3-person household co-resident multimorbidity	53.12 (52.63-53.62)	0.50 (0.50-0.51)	0.86 (0.85-0.87)	0.92 (0.91-0.93)
Age group				
18-30	49.47 (49.18-49.76)	0.77 (0.76-0.78)	0.78 (0.78-0.79)	1.05 (1.04-1.06)
30-45	43.09 (42.71-43.48)	0.71 (0.71-0.72)	0.74 (0.73-0.75)	0.90 (0.89-0.91)
46-65	38.4 (38.1-38.7)	Reference (1.00)	Reference (1.00)	Reference (1.00)
66-85	113.32 (112.71-113.93)	2.54 (2.52-2.56)	2.41 (2.39-2.43)	1.77 (1.76-1.78)
86+	285.59 (282.52-288.68)	7.05 (6.98-7.13)	6.43 (6.36-6.50)	4.06 (4.01-4.11)
Sex				
Men	58.52 (58.24-58.79)	Reference (1.00)	Reference (1.00)	Reference (1.00)
Women	64.55 (64.27-64.82)	1.02 (1.02-1.03)	0.95 (0.95-0.96)	1.09 (1.08-1.10)
Socioeconomic position				
1 (lowest)	73.39 (72.89-73.89)	Reference (1.00)	Reference (1.00)	Reference (1.00)
2	67.65 (67.2-68.11)	0.95 (0.94-0.96)	0.90 (0.89-0.91)	0.93 (0.92-0.95)
3	61.02 (60.59-61.44)	0.86 (0.85-0.87)	0.81 (0.80-0.81)	0.86 (0.85-0.87)
4	57.02 (56.59-57.45)	0.82 (0.81-0.82)	0.75 (0.74-0.75)	0.81 (0.80-0.82)
5 (highest)	51.32 (50.94-51.7)	0.74 (0.73-0.75)	0.68 (0.67-0.68)	0.74 (0.73-0.75)
Number of long-term conditions				
0-1	36.62 (36.44-36.81)	Reference (1.00)	NA	Reference (1.00)
2-3	78.8 (78.32-79.29)	2.27 (2.26-2.29)	NA	1.93 (1.91-1.95)
4-5	140.62 (139.56-141.68)	4.11 (4.08-4.15)	NA	3.16 (3.11-3.20)
6+	260.78 (258.71-262.86)	7.44 (7.38-7.51)	NA	5.41 (5.30-5.52)
Smoking			NA	
Never smoker	53.3 (53.06-53.54)	Reference (1.00)		Reference (1.00)
Ex-smoker	77.59 (77.18-78)	1.44 (1.43-1.45)	NA	1.24 (1.23-1.26)
Light smoker	65.79 (64.55-67.06)	1.18 (1.16-1.20)	NA	1.35 (1.32-1.38)
Moderate smoker	68.15 (66.99-69.33)	1.20 (1.18-1.22)	NA	1.39 (1.36-1.41)
Heavy smoker	83.29 (81.22-85.38)	1.48 (1.44-1.52)	NA	1.55 (1.51-1.59)
Missing	57.36 (56.7-58.03)	1.25 (1.23-1.26)	NA	1.34 (1.32-1.36)
Alcohol			NA	
None	84.26 (83.64-84.88)	Reference (1.00)		Reference (1.00)
Light (1-2u/day	56.4 (56.1-56.71)	0.70 (0.70-0.71)	NA	0.86 (0.85-0.87)
Moderate (3-6u/day)	51.44 (50.84-52.03)	0.65 (0.64-0.66)	NA	0.86 (0.84-0.87)
Heavy (7-9u/day)	71.72 (69.29-74.21)	0.87 (0.84-0.90)	NA	1.08 (1.04-1.13)
Very heavy (9+u/day)	88.56 (85.47-91.71)	1.00 (0.97-1.04)	NA	1.28 (1.23-1.33)
Missing	60.62 (60.31-60.93)	0.80 (0.79-0.81)	NA	0.90 (0.89-0.91)

Supplementary Table S10. Associations between all covariates with a transition to living in a care home for all variables.

Event rate per 1000 person-years and hazard ratios (HRs) with 95% confidence intervals (95%CIs) for unadjusted, partially adjusted, and fully adjusted models. The partially adjusted model incorporates age group, sex, and socioeconomic position. The fully adjusted model incorporates age group, sex, socioeconomic position, number of long-term conditions, body mass index, and alcohol consumption. Note: the inclusion of smoking in this model did not improve model fit as measured using the AIC.

**Covariate**	**Event rate/1000 person years**	**Unadjusted HR (95% CI)**	**Partially adjusted HR (95% CI)***	**Fully adjusted HR (95% CI)****
Household size & co-resident multimorbidity				
Lives alone	5.88 (5.76-6)	Reference (1.00)	Reference (1.00)	Reference (1.00)
2-person household co-resident no multimorbidity	0.70 (0.66-0.74)	0.30 (0.29-0.30)	0.62 (0.61-0.64)	0.71 (0.69-0.72)
2-person household co-resident multimorbidity	2.82 (2.73-2.92)	0.76 (0.75-0.77)	0.75 (0.74-0.76)	0.78 (0.77-0.79)
≥3-person household co-resident no multimorbidity	0.14 (0.12-0.16)	0.08 (0.08-0.08)	0.49 (0.47-0.50)	0.57 (0.55-0.59)
≥3-person household co-resident multimorbidity	0.73 (0.68-0.78)	0.23 (0.23-0.24)	0.73 (0.71-0.75)	0.78 (0.75-0.80)
Age group				
18-30	0.24 (0.22-0.26)	Reference (1.00)	Reference (1.00)	Reference (1.00)
30-45	0.06 (0.05-0.07)	0.07 (0.07-0.08)	0.11 (0.10-0.12)	0.10 (0.09-0.10)
46-65	0.05 (0.04-0.06)	0.19 (0.18-0.20)	0.27 (0.26-0.28)	0.26 (0.25-0.27)
66-85	4.54 (4.43-4.65)	6.04 (5.93-6.15)	3.70 (3.63-3.77)	3.61 (3.54-3.68)
86+	31.92 (31.15-32.69)	26.34 (25.79-26.89)	12.93 (12.62-13.24)	11.25 (10.98-11.52)
Sex				
Men	1.39 (1.35-1.43)	Reference (1.00)	Reference (1.00)	Reference (1.00)
Women	2.43 (2.38-2.48)	0.88 (0.87-0.89)	0.70 (0.69-0.71)	0.65 (0.64-0.66)
Socioeconomic position				
1 (lowest)	1.88 (1.81-1.95)	Reference (1.00)	Reference (1.00)	Reference (1.00)
2	2.05 (1.98-2.12)	1.01 (0.99-1.03)	0.94 (0.92-0.96)	0.94 (0.92-0.96)
3	1.98 (1.91-2.05)	0.91 (0.89-0.93)	0.85 (0.83-0.87)	0.86 (0.85-0.88)
4	1.94 (1.87-2.01)	0.88 (0.86-0.90)	0.80 (0.78-0.82)	0.82 (0.80-0.84)
5 (highest)	1.85 (1.78-1.92)	0.79 (0.78-0.81)	0.73 (0.71-0.74)	0.74 (0.72-0.75)
Number of long-term conditions				
0-1	0.31 (0.29-0.33)	Reference (1.00)	NA	Reference (1.00)
2-3	2.27 (2.2-2.34)	4.86 (4.76-4.96)	NA	2.06 (2.02-2.11)
4-5	5.93 (5.75-6.11)	11.66 (11.42-11.90)	NA	3.31 (3.24-3.39)
6+	12.42 (12.08-12.76)	26.61 (26.09-27.15)	NA	6.00 (5.86-6.15)
Body mass index				
18.5-24.9	2.74 (2.66-2.82)	Reference (1.00)	NA	Reference (1.00)
<18.5	5.05 (4.66-5.44)	1.85 (1.79-1.92)	NA	2.05 (1.98-2.13)
25-29.9	2.12 (2.05-2.19)	0.89 (0.88-0.91)	NA	0.70 (0.69-0.71)
30-39.9	1.46 (1.39-1.53)	0.79 (0.78-0.81)	NA	0.65 (0.64-0.66)
40+	0.98 (0.85-1.11)	0.74 (0.71-0.77)	NA	0.85 (0.81-0.88)
Missing	1.52 (1.47-1.57)	0.62 (0.61-0.63)	NA	1.06 (1.04-1.08)
Alcohol				
None	3.65 (3.53-3.77)	Reference (1.00)	NA	Reference (1.00)
Light (1-2u/day	1.35 (1.31-1.39)	0.62 (0.61-0.64)	NA	0.95 (0.94-0.97)
Moderate (3-6u/day)	0.67 (0.61-0.73)	0.56 (0.54-0.57)	NA	1.02 (0.99-1.05)
Heavy (7-9u/day)	0.57 (0.37-0.77)	0.79 (0.73-0.85)	NA	1.36 (1.26-1.47)
Very heavy (9+u/day)	0.66 (0.42-0.9)	0.82 (0.76-0.89)	NA	1.68 (1.55-1.83)
Missing	2.19 (2.14-2.24)	0.91 (0.90-0.93)	NA	1.42 (1.39-1.44)

Supplementary Table S11. Subgroup analysis 1: age stratification of unplanned hospitalisation models.

Associations between all covariates and first unplanned hospitalisation for all variables in two subgroups of the study population: those aged 65 years and younger, and those aged over 65 years. Hazard ratios (HRs) with 95% confidence intervals (95% CIs) for unadjusted and fully adjusted models. The fully adjusted model incorporates age group, sex, socioeconomic position, number of long-term conditions, smoking, and alcohol consumption.

**Covariate**	**65 years and under Unadjusted HR (95% CI)**	**65 years and under Fully adjusted HR (95% CI)**	**Over 65 years Unadjusted HR (95% CI)**	**Over 65 years Fully adjusted HR (95% CI)**
Household size & co-resident multimorbidity				
Lives alone	Reference (1.00)	Reference (1.00)	Reference (1.00)	Reference (1.00)
2-person household co-resident no multimorbidity	0.82 (0.81-0.83)	0.94 (0.93-0.95)	0.64 (0.63-0.64)	0.80 (0.79-0.81)
2-person household co-resident multimorbidity	1.01 (1.00-1.03)	0.94 (0.93-0.96)	0.73 (0.72-0.73)	0.86 (0.86-0.87)
≥3-person household co-resident no multimorbidity	0.69 (0.68-0.70)	0.90 (0.89-0.91)	0.77 (0.75-0.79)	0.90 (0.87-0.92)
≥3-person household co-resident multimorbidity	0.81 (0.80-0.82)	0.95 (0.93-0.96)	0.80 (0.78-0.81)	0.91 (0.89-0.93)
Age group				
18-30	NA	1.14 (1.13-1.16)	NA	NA
30-45	NA	0.95 (0.94-0.96)	NA	NA
46-65	NA	Reference (1.00)	NA	NA
66-85	NA	NA	NA	Reference (1.00)
86+	NA	NA	NA	2.30 (2.27-2.32)
Sex				
Men	Reference (1.00)	Reference (1.00)	Reference (1.00)	Reference (1.00)
Women	1.11 (1.10-1.11)	1.09 (1.08-1.10)	0.89 (0.89-0.90)	0.81 (0.81-0.82)
Socioeconomic position				
1 (lowest)	Reference (1.00)	Reference (1.00)	Reference (1.00)	Reference (1.00)
2	0.87 (0.86-0.88)	0.93 (0.92-0.95)	0.94 (0.92-0.95)	0.97 (0.96-0.99)
3	0.77 (0.76-0.78)	0.86 (0.85-0.87)	0.84 (0.83-0.86)	0.92 (0.91-0.94)
4	0.70 (0.69-0.71)	0.81 (0.80-0.82)	0.79 (0.78-0.80)	0.89 (0.88-0.90)
5 (highest)	0.61 (0.60-0.61)	0.74 (0.73-0.75)	0.74 (0.73-0.75)	0.85 (0.84-0.87)
Number of long-term conditions				
0-1	Reference (1.00)	Reference (1.00)	Reference (1.00)	Reference (1.00)
2-3	1.99 (1.97-2.01)	1.93 (1.91-1.95)	1.51 (1.49-1.53)	1.55 (1.52-1.57)
4-5	3.41 (3.37-3.46)	3.16 (3.11-3.20)	2.26 (2.23-2.29)	2.20 (2.17-2.23)
6+	6.11 (6.00-6.23)	5.41 (5.30-5.52)	3.69 (3.64-3.75)	3.38 (3.33-3.43)
Smoking				
Never smoker	Reference (1.00)	Reference (1.00)	Reference (1.00)	Reference (1.00)
Ex-smoker	1.46 (1.45-1.47)	1.24 (1.23-1.26)	1.17 (1.16-1.19)	1.12 (1.11-1.13)
Light smoker	1.53 (1.50-1.57)	1.35 (1.32-1.38)	1.27 (1.23-1.32)	1.31 (1.26-1.36)
Moderate smoker	1.66 (1.63-1.69)	1.39 (1.36-1.41)	1.26 (1.22-1.31)	1.32 (1.28-1.37)
Heavy smoker	2.09 (2.04-2.15)	1.55 (1.51-1.59)	1.52 (1.44-1.60)	1.51 (1.44-1.59)
Missing	1.28 (1.26-1.30)	1.34 (1.32-1.36)	1.55 (1.52-1.58)	1.87 (1.83-1.90)
Alcohol				
None	Reference (1.00)	Reference (1.00)	Reference (1.00)	Reference (1.00)
Light (1-2u/day	0.71 (0.70-0.72)	0.86 (0.85-0.87)	0.88 (0.87-0.89)	0.96 (0.95-0.97)
Moderate (3-6u/day)	0.73 (0.72-0.75)	0.86 (0.84-0.87)	0.86 (0.84-0.87)	0.94 (0.92-0.96)
Heavy (7-9u/day)	1.11 (1.07-1.16)	1.08 (1.04-1.13)	1.08 (1.01-1.15)	1.08 (1.02-1.15)
Very heavy (9+u/day)	1.45 (1.40-1.51)	1.28 (1.23-1.33)	1.09 (1.00-1.19)	1.06 (0.98-1.16)
Missing	0.74 (0.73-0.74)	0.90 (0.89-0.91)	1.14 (1.13-1.15)	1.18 (1.16-1.19)

Supplementary Table S12. Subgroup analysis 2: age stratification of care home models.

Associations between all covariates with time to transitioning to live in a care home for all variables in two subgroups of the study population a. those aged 65 years and younger, and b. those aged over 65 years. Hazard ratios (HRs) with 95% confidence intervals (95%CIs) for unadjusted and fully adjusted models. The fully adjusted model incorporates age group, sex, socioeconomic position, number of long-term conditions, body mass index, and alcohol consumption.

**Covariate**	**65 years and under Unadjusted HR (95% CI)**	**65 years and under Fully adjusted HR (95% CI)**	**Over 65 years Unadjusted HR (95% CI)**	**Over 65 years Fully adjusted HR (95% CI)**
Household size & co-resident multimorbidity				
Lives alone	Reference (1.00)	Reference (1.00)	Reference (1.00)	Reference (1.00)
2-person household co-resident no multimorbidity	0.49 (0.47-0.51)	0.67 (0.64-0.70)	0.50 (0.49-0.51)	0.71 (0.70-0.73)
2-person household co-resident multimorbidity	0.98 (0.94-1.02)	0.78 (0.75-0.81)	0.61 (0.60-0.62)	0.78 (0.77-0.79)
≥3-person household co-resident no multimorbidity	0.22 (0.21-0.23)	0.47 (0.45-0.49)	0.70 (0.67-0.73)	0.89 (0.85-0.93)
≥3-person household co-resident multimorbidity	0.42 (0.40-0.44)	0.65 (0.62-0.68)	0.71 (0.69-0.74)	0.86 (0.84-0.89)
Age group				
18-30	NA	0.13 (0.12-0.14)	NA	NA
30-45	NA	0.32 (0.31-0.34)	NA	NA
46-65	NA	Reference (1.00)	NA	NA
66-85	NA	NA	NA	Reference (1.00)
86+	NA	NA	NA	3.21 (3.15-3.27)
Sex				
Men	Reference (1.00)	Reference (1.00)	Reference (1.00)	Reference (1.00)
Women	0.68 (0.66-0.70)	0.64 (0.62-0.66)	0.85 (0.84-0.86)	0.66 (0.65-0.67)
Socioeconomic position				
1 (lowest)	Reference (1.00)	Reference (1.00)	Reference (1.00)	Reference (1.00)
2	0.82 (0.79-0.86)	0.91 (0.88-0.95)	0.93 (0.91-0.95)	0.96 (0.94-0.98)
3	0.71 (0.68-0.74)	0.84 (0.80-0.88)	0.81 (0.79-0.83)	0.88 (0.86-0.91)
4	0.64 (0.61-0.67)	0.80 (0.76-0.84)	0.75 (0.73-0.77)	0.84 (0.82-0.86)
5 (highest)	0.53 (0.50-0.55)	0.70 (0.66-0.73)	0.69 (0.67-0.70)	0.76 (0.74-0.78)
Number of long-term conditions				
0-1	Reference (1.00)	Reference (1.00)	Reference (1.00)	Reference (1.00)
2-3	3.86 (3.73-4.00)	2.73 (2.63-2.84)	1.57 (1.53-1.61)	1.62 (1.58-1.66)
4-5	9.15 (8.78-9.54)	5.46 (5.21-5.71)	2.54 (2.47-2.60)	2.52 (2.46-2.59)
6+	21.45 (20.49-22.45)	11.57 (10.99-12.18)	4.85 (4.74-4.97)	4.60 (4.48-4.72)
Body mass index				
18.5-24.9	Reference (1.00)	Reference (1.00)	Reference (1.00)	Reference (1.00)
<18.5	2.13 (1.96-2.32)	2.41 (2.21-2.63)	2.05 (1.97-2.13)	1.96 (1.88-2.04)
25-29.9	1.09 (1.04-1.14)	0.70 (0.67-0.73)	0.68 (0.67-0.70)	0.70 (0.69-0.72)
30-39.9	1.34 (1.28-1.40)	0.64 (0.61-0.67)	0.63 (0.62-0.65)	0.64 (0.63-0.66)
40+	2.04 (1.91-2.19)	0.83 (0.77-0.89)	0.75 (0.71-0.79)	0.78 (0.74-0.83)
Missing	0.88 (0.84-0.92)	0.98 (0.94-1.03)	0.84 (0.83-0.86)	1.08 (1.06-1.11)
Alcohol				
None	Reference (1.00)	Reference (1.00)	Reference (1.00)	Reference (1.00)
Light (1-2u/day	0.66 (0.63-0.70)	0.92 (0.88-0.97)	0.87 (0.85-0.89)	0.97 (0.95-1.00)
Moderate (3-6u/day)	1.02 (0.96-1.08)	1.11 (1.05-1.18)	0.84 (0.81-0.87)	0.99 (0.96-1.03)
Heavy (7-9u/day)	2.01 (1.80-2.26)	1.54 (1.37-1.73)	1.12 (1.01-1.25)	1.18 (1.06-1.32)
Very heavy (9+u/day)	2.74 (2.46-3.04)	1.91 (1.71-2.13)	1.12 (0.97-1.29)	1.18 (1.02-1.37)
Missing	0.95 (0.91-1.00)	1.54 (1.47-1.61)	1.33 (1.31-1.36)	1.39 (1.36-1.42)

Supplementary Table S13. Subgroup analysis 3: sex stratification of unplanned hospitalisation models.

Associations between this variable with first unplanned hospitalisation: hazard ratios (HRs) with 95% confidence intervals (95%CIs) for unadjusted and fully adjusted models. The fully adjusted model for time to the first unplanned hospitalisation outcome incorporates age group, sex, socioeconomic position, number of long-term conditions, smoking, and alcohol consumption.

**Covariate**	**Men Unadjusted HR (95% CI)**	**Men Fully adjusted HR (95% CI)**	**Women Unadjusted HR (95% CI)**	**Women Fully adjusted HR (95% CI)**
Household size & co-resident multimorbidity				
Lives alone	Reference (1.00)	Reference (1.00)	Reference (1.00)	Reference (1.00)
2-person household co-resident no multimorbidity	0.63 (0.62-0.64)	0.83 (0.82-0.85)	0.49 (0.48-0.49)	0.91 (0.90-0.92)
2-person household co-resident multimorbidity	1.13 (1.12-1.15)	0.90 (0.89-0.91)	0.74 (0.73-0.75)	0.89 (0.88-0.90)
≥3-person household co-resident no multimorbidity	0.38 (0.38-0.39)	0.82 (0.81-0.83)	0.37 (0.36-0.37)	0.88 (0.86-0.89)
≥3-person household co-resident multimorbidity	0.56 (0.55-0.57)	0.92 (0.91-0.94)	0.50 (0.49-0.50)	0.92 (0.91-0.94)
Age group				
18-30	0.56 (0.55-0.56)	0.74 (0.72-0.75)	1.01 (0.99-1.02)	1.38 (1.36-1.40)
30-45	0.60 (0.59-0.61)	0.75 (0.74-0.76)	0.83 (0.82-0.84)	1.06 (1.04-1.07)
46-65		Reference (1.00)		NA
66-85	2.57 (2.54-2.59)	1.80 (1.78-1.82)	2.46 (2.44-2.49)	1.75 (1.73-1.77)
86+	7.03 (6.92-7.15)	4.15 (4.08-4.23)	6.90 (6.81-7.00)	4.04 (3.98-4.11)
Socioeconomic position				
1 (lowest)	Reference (1.00)	Reference (1.00)	Reference (1.00)	Reference (1.00)
2	0.96 (0.94-0.97)	0.96 (0.94-0.97)	0.95 (0.93-0.96)	0.95 (0.94-0.96)
3	0.89 (0.87-0.90)	0.89 (0.88-0.91)	0.85 (0.84-0.86)	0.89 (0.88-0.90)
4	0.85 (0.83-0.86)	0.85 (0.84-0.87)	0.80 (0.79-0.81)	0.85 (0.83-0.86)
5 (highest)	0.78 (0.77-0.79)	0.80 (0.79-0.81)	0.73 (0.72-0.73)	0.79 (0.78-0.80)
Number of long-term conditions				
0-1	Reference (1.00)	Reference (1.00)	Reference (1.00)	Reference (1.00)
2-3	2.53 (2.50-2.56)	1.80 (1.78-1.82)	2.08 (2.06-2.10)	1.79 (1.77-1.81)
4-5	4.56 (4.50-4.62)	2.60 (2.56-2.63)	3.79 (3.74-3.83)	2.64 (2.61-2.68)
6+	8.19 (8.08-8.30)	3.95 (3.89-4.01)	6.91 (6.83-6.99)	4.05 (3.99-4.11)
Smoking				
Never smoker	Reference (1.00)	Reference (1.00)	Reference (1.00)	Reference (1.00)
Ex-smoker	1.68 (1.66-1.70)	1.16 (1.15-1.17)	1.26 (1.25-1.27)	1.18 (1.17-1.19)
Light smoker	1.29 (1.25-1.32)	1.37 (1.33-1.41)	1.11 (1.08-1.14)	1.30 (1.27-1.33)
Moderate smoker	1.29 (1.26-1.33)	1.42 (1.38-1.45)	1.15 (1.12-1.18)	1.34 (1.31-1.37)
Heavy smoker	1.59 (1.54-1.65)	1.54 (1.49-1.59)	1.45 (1.40-1.50)	1.53 (1.48-1.58)
Missing	1.26 (1.24-1.28)	1.52 (1.49-1.54)	1.30 (1.27-1.32)	1.57 (1.55-1.60)
Alcohol				
None	Reference (1.00)	Reference (1.00)	Reference (1.00)	Reference (1.00)
Light (1-2u/day	0.72 (0.71-0.73)	0.91 (0.90-0.92)	0.67 (0.66-0.67)	0.92 (0.91-0.93)
Moderate (3-6u/day)	0.63 (0.62-0.64)	0.90 (0.89-0.92)	0.61 (0.60-0.63)	0.91 (0.89-0.94)
Heavy (7-9u/day)	0.82 (0.79-0.85)	1.09 (1.05-1.13)	0.91 (0.85-0.98)	1.16 (1.09-1.25)
Very heavy (9+u/day)	0.94 (0.91-0.98)	1.28 (1.23-1.33)	1.01 (0.94-1.08)	1.25 (1.17-1.34)
Missing	0.68 (0.68-0.69)	1.03 (1.02-1.05)	0.88 (0.88-0.89)	1.06 (1.05-1.07)

Supplementary Table S14. Subgroup analysis 4: sex stratification of care home models.

Associations between this variable with the transition to living in a care home: hazard ratios (HRs) with 95% confidence intervals (95%CIs) for unadjusted and fully adjusted models. The fully adjusted model for time to transition to live in a care home incorporates age group, sex, socioeconomic position, number of long-term conditions, body mass index, and alcohol consumption.

**Covariate**	**Men Unadjusted HR (95% CI)**	**Men Fully adjusted HR (95% CI)**	**Women Unadjusted HR (95% CI)**	**Women Fully adjusted HR (95% CI)**
Household size & co-resident multimorbidity				
Lives alone	Reference (1.00)	Reference (1.00)	Reference (1.00)	Reference (1.00)
2-person household co-resident no multimorbidity	0.41 (0.40-0.42)	0.68 (0.66-0.70)	0.21 (0.21-0.22)	0.77 (0.75-0.80)
2-person household co-resident multimorbidity	1.10 (1.08-1.12)	0.79 (0.77-0.81)	0.58 (0.56-0.59)	0.79 (0.77-0.81)
≥3-person household co-resident no multimorbidity	0.10 (0.10-0.10)	0.52 (0.50-0.54)	0.06 (0.06-0.06)	0.66 (0.63-0.70)
≥3-person household co-resident multimorbidity	0.28 (0.27-0.29)	0.72 (0.69-0.75)	0.22 (0.21-0.23)	0.87 (0.84-0.91)
Age group				
18-30	0.09 (0.08-0.10)	0.11 (0.10-0.12)	0.06 (0.05-0.06)	0.07 (0.06-0.09)
30-45	0.20 (0.19-0.22)	0.27 (0.26-0.29)	0.18 (0.17-0.19)	0.25 (0.23-0.27)
46-65	Reference (1.00)	Reference (1.00)	Reference (1.00)	Reference (1.00)
66-85	5.84 (5.70-5.98)	3.44 (3.35-3.54)	6.43 (6.26-6.60)	3.87 (3.75-3.98)
86+	25.82 (25.04-26.63)	10.77 (10.39-11.16)	30.41 (29.53-31.31)	12.68 (12.24-13.13)
Socioeconomic position				
1 (lowest)	Reference (1.00)	Reference (1.00)	Reference (1.00)	Reference (1.00)
2	0.98 (0.95-1.01)	0.94 (0.91-0.97)	1.03 (1.01-1.06)	0.95 (0.92-0.97)
3	0.89 (0.87-0.92)	0.85 (0.83-0.88)	0.93 (0.91-0.96)	0.87 (0.85-0.90)
4	0.86 (0.84-0.89)	0.81 (0.78-0.83)	0.90 (0.87-0.92)	0.83 (0.80-0.85)
5 (highest)	0.79 (0.77-0.82)	0.73 (0.71-0.76)	0.81 (0.79-0.83)	0.74 (0.71-0.76)
Number of long-term conditions				
0-1	Reference (1.00)	Reference (1.00)	Reference (1.00)	Reference (1.00)
2-3	4.91 (4.78-5.05)	2.07 (2.01-2.14)	4.99 (4.85-5.14)	2.03 (1.97-2.10)
4-5	11.36 (11.04-11.69)	3.34 (3.23-3.45)	12.56 (12.20-12.93)	3.29 (3.18-3.40)
6+	26.31 (25.59-27.05)	6.29 (6.09-6.51)	28.75 (27.94-29.58)	5.93 (5.74-6.14)
Body mass index				
18.5-24.9	Reference (1.00)	Reference (1.00)	Reference (1.00)	Reference (1.00)
<18.5	1.46 (1.37-1.55)	2.43 (2.26-2.60)	2.20 (2.11-2.30)	2.07 (1.98-2.18)
25-29.9	0.76 (0.74-0.78)	0.66 (0.64-0.68)	0.95 (0.93-0.97)	0.73 (0.71-0.75)
30-39.9	0.68 (0.66-0.70)	0.60 (0.58-0.62)	0.86 (0.84-0.89)	0.69 (0.67-0.71)
40+	0.68 (0.63-0.73)	0.77 (0.72-0.83)	0.84 (0.80-0.88)	0.91 (0.86-0.96)
Missing	0.37 (0.36-0.37)	0.92 (0.89-0.94)	0.95 (0.93-0.97)	1.20 (1.17-1.23)
Alcohol				
None	Reference (1.00)	Reference (1.00)	Reference (1.00)	Reference (1.00)
Light (1-2u/day	0.67 (0.65-0.69)	0.94 (0.92-0.97)	0.54 (0.53-0.56)	0.96 (0.94-0.99)
Moderate (3-6u/day)	0.53 (0.51-0.55)	1.04 (1.00-1.08)	0.39 (0.36-0.41)	1.00 (0.93-1.07)
Heavy (7-9u/day)	0.72 (0.66-0.78)	1.34 (1.23-1.47)	0.64 (0.53-0.77)	1.53 (1.26-1.87)
Very heavy (9+u/day)	0.77 (0.70-0.84)	1.70 (1.54-1.87)	0.63 (0.51-0.76)	1.67 (1.36-2.06)
Missing	0.73 (0.71-0.75)	1.46 (1.42-1.50)	1.02 (1.00-1.05)	1.39 (1.36-1.43)

Supplementary Table S15. Sensitivity analyses 5: household size and household co-resident mental-physical multimorbidity.

Associations between a. first unplanned hospitalisation and b. transition to living in a care home for all variables: hazard ratios (HRs) with 95% confidence intervals (95%CIs) for unadjusted and fully adjusted models. The fully adjusted model for time to transition to live in a care home incorporates age group, sex, socioeconomic position, number of long-term conditions, body mass index, and alcohol consumption. Note that unadjusted coefficients for each covariate are not included here as they are the same as those presented in Table 2 of the main manuscript.

**Covariate**	**Unplanned hospitalisation Unadjusted HR (95% CI)**	**Unplanned hospitalisation Fully adjusted HR (95% CI)**	**Transition to care home Unadjusted HR (95% CI)**	**Transition to care home Fully adjusted HR (95% CI)**
Household size & co-resident mental-physical multimorbidity				
Lives alone	Reference (1.00)	Reference (1.00)	Reference (1.00)	Reference (1.00)
2-person household co-resident no mental-physical multimorbidity	0.47 (0.46-0.48)	0.90 (0.89-0.91)	0.67 (0.66-0.67)	0.75 (0.74-0.76)
2-person household co-resident mental-physical multimorbidity	0.70 (0.69-0.72)	0.93 (0.92-0.94)	0.86 (0.85-0.87)	0.76 (0.74-0.78)
≥3-person household co-resident no mental-physical multimorbidity	0.11 (0.11-0.11)	0.88 (0.87-0.89)	0.39 (0.39-0.40)	0.66 (0.64-0.67)
≥3-person household co-resident mental-physical multimorbidity	0.22 (0.22-0.23)	0.96 (0.94-0.97)	0.50 (0.50-0.51)	0.80 (0.77-0.83)

Supplementary Table S16. Sensitivity analyses 6: household size and household co-resident number of long-term conditions.

Associations between a. first unplanned hospitalisation and b. transition to living in a care home for all variables: hazard ratios (HRs) with 95% confidence intervals (95%CIs) for unadjusted and fully adjusted models. In three-or-more-person households, the level refers to the highest number of LTCs in any co-resident. The fully adjusted model for time to the first unplanned hospitalisation outcome incorporates age group, sex, socioeconomic position, number of long-term conditions, smoking, and alcohol consumption. The fully adjusted model for time to transition to live in a care home incorporates age group, sex, socioeconomic position, number of long-term conditions, body mass index, and alcohol consumption. Note that unadjusted coefficients for each covariate are not included here as they are the same as those presented in Table 2 of the main manuscript.

**Covariate**	**Unplanned hospitalisation Unadjusted HR (95% CI)**	**Unplanned hospitalisation Fully adjusted HR (95% CI)**	**Transition to care home Unadjusted HR (95% CI)**	**Transition to care home Fully adjusted HR (95% CI)**
Household size & co-resident LTCs				
Lives alone	Reference (1.00)	Reference (1.00)	Reference (1.00)	Reference (1.00)
2-person household co-resident 0-1 LTCs	0.68 (0.68-0.69)	0.90 (0.89-0.91)	0.50 (0.49-0.50)	0.76 (0.74-0.77)
2-person household co-resident 2-3 LTCs	0.69 (0.68-0.70)	0.91 (0.90-0.92)	0.50 (0.49-0.52)	0.76 (0.74-0.78)
2-person household co-resident 4+ LTCs	0.72 (0.71-0.73)	0.94 (0.92-0.95)	0.50 (0.49-0.52)	0.75 (0.72-0.77)
≥3-person household co-resident 0-1 LTCs	0.40 (0.40-0.40)	0.89 (0.88-0.90)	0.12 (0.12-0.13)	0.68 (0.66-0.70)
≥3-person household co-resident 2-3 LTCs	0.40 (0.40-0.41)	0.89 (0.88-0.90)	0.13 (0.12-0.13)	0.70 (0.67-0.73)
≥3-person household co-resident 4+ LTCs	0.43 (0.42-0.43)	0.95 (0.93-0.96)	0.13 (0.12-0.13)	0.69 (0.65-0.72)

References

1. Ho IS-S, Azcoaga-Lorenzo A, Akbari A, et al. Examining variation in the measurement of multimorbidity in research: a systematic review of 566 studies. *The Lancet Public Health* 2021;6(8):e587-e97. doi: 10.1016/s2468-2667(21)00107-9
2. Ford DV, Jones KH, Verplancke J-P, et al. The SAIL Databank: Building a national architecture for e-health research and evaluation. *BMC Health Serv Res* 2009;9(1):157-57. doi: 10.1186/1472-6963-9-157
3. MacRae C, Mercer SW, Henderson D, et al. Age, sex, and socioeconomic differences in multimorbidity measured in four ways: UK primary care cross-sectional analysis. *Br J Gen Pract* 2023:BJGP.2022.0405. doi: 10.3399/BJGP.2022.0405
4. Welsh Longitudonal General Practice Dataset (WLGP) - Welsh Primary Care. Available at https://web.www.healthdatagateway.org/dataset/33fc3ffd-aa4c-4a16-a32f-0c900aaea3d2#:~:text=This%20dataset%20covers%2083%25%20of,including%20bespoke%20project%20specific%20cohorts accessed 29 January 2024 [
5. MacRae C, Morales D, Mercer SW, et al. Impact of data source choice on multimorbidity measurement: a comparison study of 2.3 million individuals in the Welsh National Health Service. *BMC Med* 2023;21(1):1-309. doi: 10.1186/s12916-023-02970-z
6. Ho ISS, Azoaga-Lorenzo A, Akbari A, Davies J, Khunti K, Kadam U, et al Measuring multimorbidity in research: a Delphi consensus study. *BMJ Medicine* July 2022 doi: http://dx.doi.org/10.1136/bmjmed-2022-000247
7. MacRae C, McMinn M, Mercer SW, et al. The impact of varying the number and selection of conditions on estimated multimorbidity prevalence: A cross-sectional study using a large, primary care population dataset. *PLoS Med* 2023;20(4):e1004208-e08. doi: 10.1371/journal.pmed.1004208
8. Patient Episode Dataset for Wales (PEDW). Available at https://web.www.healthdatagateway.org/dataset/4c33a5d2-164c-41d7-9797-dc2b008cc852 accessed on 29 Jan 2024 [
9. MacRae C, McMinn M, Mercer SW, et al. The impact of varying the number and selection of conditions on estimated multimorbidity prevalence::a cross-sectional study using a large, primary care population dataset. 2023
10. Barnett K, Mercer SW, Norbury M, et al. Epidemiology of multimorbidity and implications for health care, research, and medical education: a cross-sectional study. *The Lancet* 2012;380(9836):37-43. doi: 10.1016/S0140-6736(12)60240-2
11. Kuan V, Denaxas S, Gonzalez-Izquierdo A, et al. A chronological map of 308 physical and mental health conditions from 4 million individuals in the English National Health Service. *The Lancet Digital health* 2019;1(2):e63-e77. doi: 10.1016/S2589-7500(19)30012-3
12. Health Data Research UK (HDRUK) Phenotype Library. Available at https://phenotypes.healthdatagateway.org/phenotypes/ [accessed 7 February 2023] [
13. Payne RA, Mendonca SC, Elliott MN, et al. Development and validation of the Cambridge Multimorbidity Score. *Canadian Medical Association journal (CMAJ)* 2020;192(5):E107-E14. doi: 10.1503/cmaj.190757
14. National Statistics Wales, Statistical Bulletin, available at https://www.gov.wales/sites/default/files/statistics-and-research/2018-12/121217sb1262012en.pdf [accessed 29 November 2023] [
15. Welsh Index of Multiple Deprivation (WIMD) 2019. Available at https://wimd.gov.wales/explore?lang=en#&domain=overall&z=8.175778660539162&lat=52.5313&lng=-4.3400 [accessed 01 August 2022] [
16. National Centre for Research Methods Working Paper 2013, Availability and use of UK based ethnicity data for health research. Available at https://eprints.ncrm.ac.uk/id/eprint/3040/1/Mathur-_Availability_and_use_of_UK_based_ethnicity_data_for_health_res_1.pdf [accessed 15 November 2023] [

17. Mathur R, Farmer RE, Eastwood SV, et al. Ethnic disparities in initiation and intensification of diabetes treatment in adults with type 2 diabetes in the UK, 1990-2017: A cohort study. *PLoS Med* 2020;17(5):e1003106. doi: 10.1371/journal.pmed.1003106
